# Implementation of a PCR-based strategy to control an outbreak by *Serratia marcescens* in a Neonatal Intensive Care Unit

**DOI:** 10.1186/s12941-023-00657-0

**Published:** 2023-12-11

**Authors:** Ángel Rodríguez-Villodres, José Manuel Ortiz de la Rosa, Raquel Valencia-Martin, Francisco Jiménez Parrilla, Guillermo Martín-Gutiérrez, Natividad Márquez Patiño, Estela Perea Cruz, María Teresa Sánchez Jiménez, Antonio Pavón Delgado, José Miguel Cisneros, José Antonio Lepe

**Affiliations:** 1 Clinical Unit of Infectious Diseases, Microbiology and Parasitology, University Hospital Virgen del Rocío, Av. Manuel Siurot s/n, 41013 Seville, Spain; 2 Institute of Biomedicine of Seville (IBiS), University Hospital Virgen del Rocío, CSIC, University of Seville, Seville, Spain; 3 Centro de Investigación Biomédica en Red de Enfermedades Infecciosas (CIBERINFEC). Instituto de Salud Carlos III, Madrid, Spain; 4 Clinical Unit of Preventive Medicine and Public Health, University Hospital Virgen del Rocío, Seville, Spain; 5 Department of Preventive Medicine, University of Seville, Seville, Spain; 6 Clinical Unit of Neonatology, University Hospital Virgen del Rocío, Seville, Spain; 7 Faculty of Medicine, University of Seville, Seville, Spain; 8 Department of Microbiology, University of Seville, Seville, Spain

**Keywords:** *Serratia marcescens*, PCR, Outbreak, Neonatal Intensive Care Unit, Epidemiology

## Abstract

**Objectives:**

To evaluate the clinical and epidemiological impact of a new molecular surveillance strategy based on qPCR to control an outbreak by *Serratia marcescens* in a Neonatal Intensive Care Unit (NICU).

**Methods:**

We design a specific qPCR for the detection of *S. marcescens* in rectal swabs of patients admitted to a NICU. We divided the surveillance study into two periods: (a) the pre-PCR, from the outbreak declaration to the qPCR introduction, and (b) the PCR period, from the introduction of the qPCR until the outbreak was solved. In all cases, *S. marcescens* isolates were recovered and their clonal relationship was analysed by PFGE. Control measures were implemented during the outbreak. Finally, the number of bloodstream infections (BSI) was investigated in order to evaluate the clinical impact of this molecular strategy.

**Results:**

Nineteen patients colonized/infected by *S. marcescens* were detected in the pre-PCR period (October 2020–April 2021). On the contrary, after the PCR implementation, 16 new patients were detected. The PFGE revealed 24 different pulsotypes belonging to 7 different clonal groups, that were not overlapping at the same time. Regarding the clinical impact, 18 months after the qPCR implementation, no more outbreaks by *S. marcescens* have been declared in the NICU of our hospital, and only 1 episode of BSI has occurred, compared with 11 BSI episodes declared previously to the outbreak control.

**Conclusions:**

The implementation of this qPCR strategy has proved to be a useful tool to control the nosocomial spread of *S. marcescens* in the NICU.

**Supplementary Information:**

The online version contains supplementary material available at 10.1186/s12941-023-00657-0.

## Introduction

*Serratia marcescens* is a gram-negative microorganism causing nosocomial infections associated with immunocompromised or critically ill patients [1]. Neonates are high-risk patients due, mainly, to the immaturity of their immune system [2]. Among the infections caused by *S. marcescens* in these patients, the most life-threatening are bloodstream infections (BSI) [3]. Furthermore, *S. marcescens* represents a high percentage (about 15%) of the microorganisms causing infections in Neonatal Intensive Care Units (NICU), due to their success in the dissemination caused by this way nosocomial outbreaks [3–7].

On 5th October 2020, after the successive detection of 5 cases of *S. marcescens*, a suspected outbreak was declared in the NICU of the University Hospital Virgen del Rocío (Seville, Spain). Two of these *S. marcescens* were isolated from BSI of 2 patients and the other 3 were isolated casually in another 3 patients from rectal swabs of the multidrug-resistant surveillance program of the hospital. In April 2021, after 14 new cases of *S. marcescens* colonization (n = 12) and infection (n = 2), a new molecular surveillance strategy based on qPCR was implemented in our hospital in order to control the recurrence/reappearance of outbreaks. Thus, the aim of this study was to evaluate the impact of this new strategy on the control of a *S. marcescens* nosocomial outbreak in a NICU through an epidemiological and microbiological investigation.

## Methods

### Surveillance strategy

To better understand the evolution of the outbreak, the surveillance strategy was divided into 2 periods: (a) the pre-PCR period encompassing from the outbreak declaration (October 2020) to 1 month before the introduction of the PCR for *S. marcescens* (March 2021), and (b) the PCR period encompassing from the introduction of the PCR (April 2021) until the resolution of the outbreak (September 2021). In both periods, rectal swabs were collected weekly from patients admitted to the NICU and processed in the Microbiology Service of the hospital. In the pre-PCR period, the surveillance strategy was based on the detection of *S. marcescens* growing the streaked agar plates used for the multidrug-resistant screening program (Brillance ESBL agar; Oxoid, UK). Instead, in the PCR period, the DNA was extracted from the rectal swabs and used for qPCR, as described below. Results were immediately informed to the Preventive Medicine Unit and the NICU team to take the appropriate control measures rapidly.

### qPCR design and analysis

Primers and probe were designed according to the previously published by Iwaya et al. [8] but with a slight modification in the probe (forward primer *Serm-F* 5′-GGT GAG CTT AA TAC GTT CAT CAA TTG-3′, reverse primer “*Serm*-R” 5′-GCA GTT CCC AGG TTG AGC C-3′ and probe *Ser-Pb* 5′-FAM-TGC GCT TTA CGC CCA GTA ATT CCG A-BHQ1-3′). This set of primers amplifies a region of 179 bp on the 16S rRNA of *S. marcescens*. PCR conditions were set as follows: 1 initial cycle at 95 °C during 5 min, and 35 cycles of 3 temperatures: 95 °C during 30 s (denaturation), 63 °C during 20 s (annealing) and 72 °C during 25 s (amplification) using the LightCycler Multiplex DNA Master Polymerase (Roche, USA). Results were visualized and analysed using the QuantStudio Design and Analysis Software v1.5.1 (Applied Biosystems, USA). To test the specificity of the primers/probe set, different strains of *S. liquefaciens*, *S. odorifera*, *Escherichia coli*, *Klebsiella pneumoniae*, *K. aerogenes*, *Pseudomonas aeruginosa*, *Morganella morganii*, *Enterobacter cloacae*, and *Proteus mirabilis* were used.

### Microbiological and epidemiological analysis

In those cases where DNA of *S. marcescens* was detected, rectal swabs were streaked onto *Salmonella*-*Shigella* agar plates with a colistin disk diffusion. Being *S. marcescens* intrinsically resistant to colistin, plates were examined after 24 h of incubation at 37 °C looking for inner colonies in the inhibition zone of the colistin disk. Identification of suspected colonies was performed by MALDI-TOF (Bruker, USA). Additionally, a Pulse Field Gel Electrophoresis (PFGE) was performed using the CHEF-DR III System (Bio-Rad, Spain) and the restriction enzyme XbaI. Analysis of the gel images was performed through the free software GelJ [9]. A dendrogram was constructed based on the Dice coefficient and 1% position tolerance. A clonal relationship was established when the similarity among the isolates was ≥ 80%, as described previously [10].

### Control measures

Comprehensive control measures were implemented in order to control and prevent *S. marcescens* transmission among the patients. A structured observation of hand hygiene compliance during the daily care activity was performed weekly by the nurses of the Preventive Medicine Unit in the NICU, following the WHO recommendations [11]. Furthermore, an exhaustive cleaning policy was instituted in order to control environmental contamination. The disinfectant used was a hypochlorite-based sanitizer; for the handling of not-disposable medical products in which this disinfectant was not appropriate, a wipe disinfectant containing benzalkonium chloride and propane-1,2-diol was used. Medical products were exclusive to each colonized or infected patient. In addition, to clarify the origin of the *S. marcescens* outbreak, several samples were taken on the different surfaces of the NICU and related dependencies, especially in the sink area, the food preparation area, and the bottle cleaning area.

### Follow-up and clinical impact

The data relating to the bloodstream infections caused by *S. marcescens* occurring in the NICU of our hospital were collected and analyzed after being divided into 2 periods: (1) from January 2019 until September 2021, when the outbreak was declared solved and (2) from October 2021 to November 2022.

## Results

**Fig. 1:**
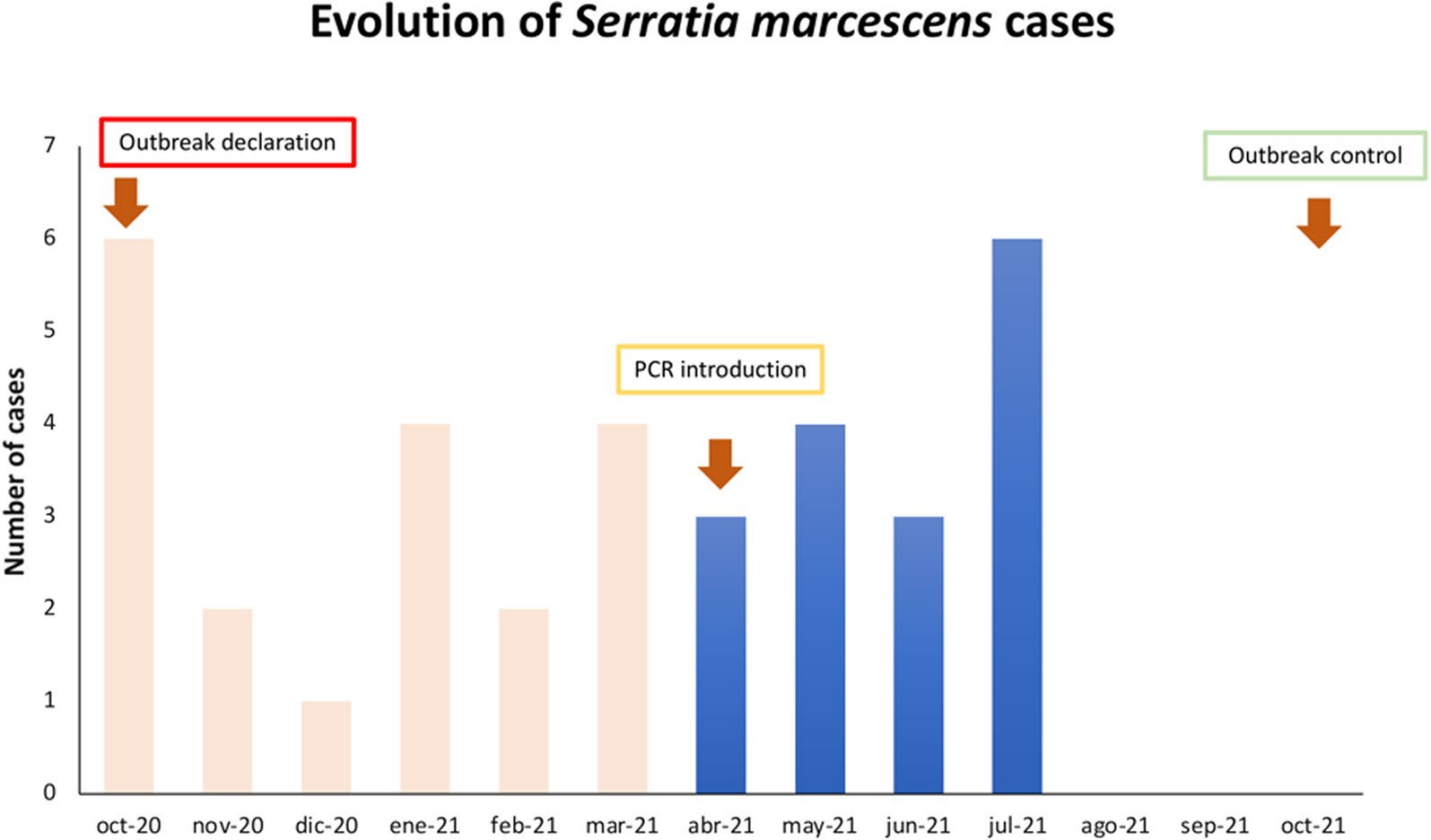
Evolution of the cases of *S. marcescens* colonization detected. *S. marcescens* isolated from culture plates (orange bars) and *S. marcescens* detected by PCR (blue bars)

From the Outbreak declaration (October 2020) until the PCR for *S. marcescens* was implemented (April 2021), a total of 19 patients colonized/infected by *S. marcescens* were detected in the NICU. On the other hand, after the implementation of the PCR, a total of 16 patients colonized by *S. marcescens* were detected by qPCR from April to the first week of July 2021 (Fig. 1). Out of them, 2 patients also suffered a sepsis episode by *S. marcescens* in this period. This qPCR allowed the detection of a higher proportion of colonized patients controlling the appearance of an outbreak. Evidence of the beneficial introduction of the *S. marcescens* qPCR was the absence of new cases of *S. marcescens* colonization/infection in September 2021, being the outbreak considered solved.

Regarding the environmental study, 231 samples were recovered and subjected to the detection of *S. marcescens* DNA. Fifteen environmental samples were positive in the qPCR assay, corresponding to 8 different surfaces of the NICU (sink, taps, milk, worktop, feeding bottles, incubator water tank, incubator surfaces, and baby bottle stopper). Finally, 6 *S. marcescens* isolates were subsequently recovered, which were included in the epidemiological analysis.

### Epidemiology

**Fig. 2:**
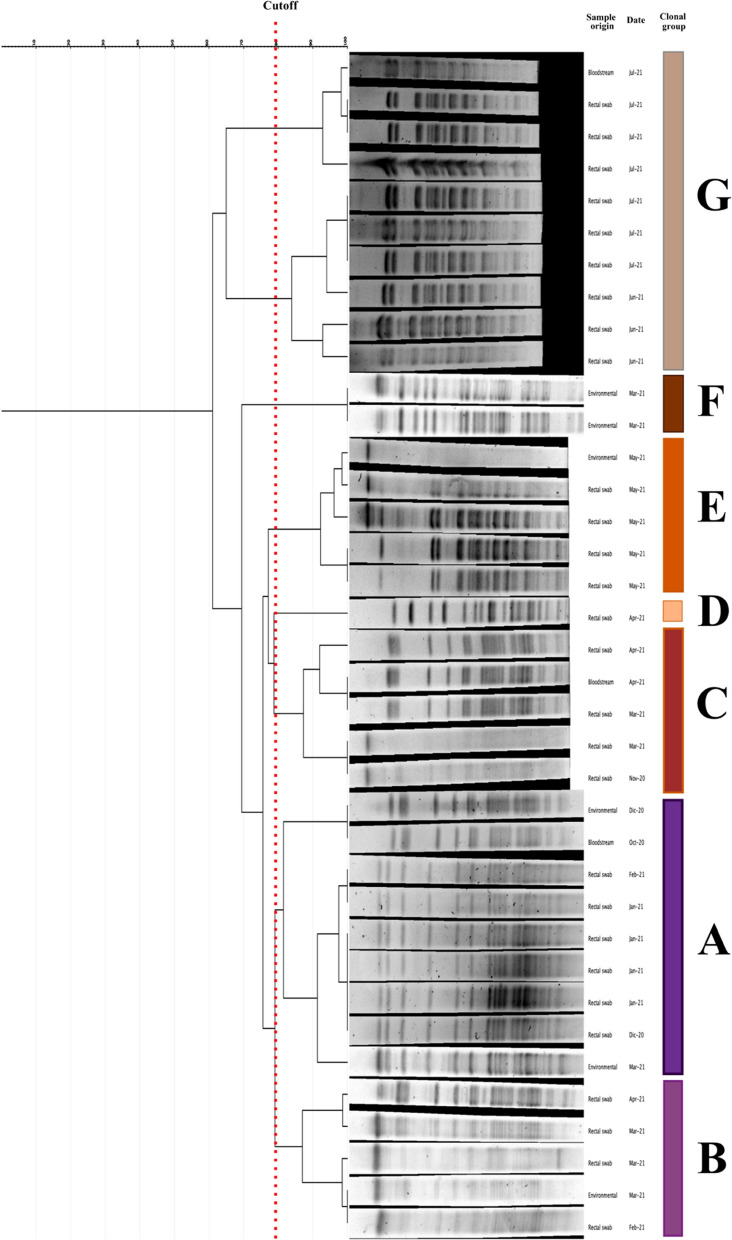
Dendrogram obtained from the *S. marcescens* isolated during the outbreak. Lanes corresponding to the isolates are cropped images from the original PFGEs. Full-length gels are presented in Additional file 1: Fig. S1

**Fig. 3:**
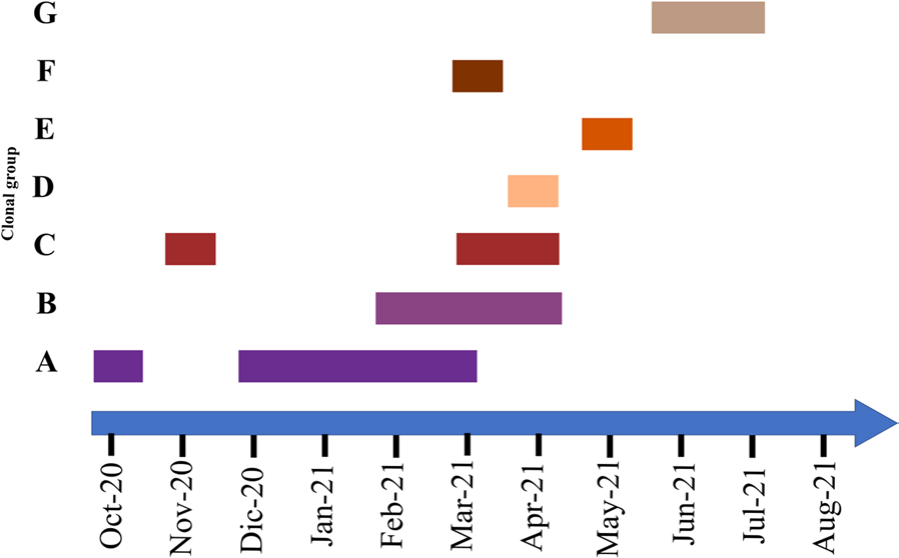
Temporary evolution of the different clonal groups from *S. marcescens* found during this outbreak

The PFGE assay performed in the 37 *S. marcescens* isolates from different sources (rectal swabs, bloodstream infection, or environmental) showed 24 different pulsotypes grouped in 7 clonal groups (A-G) (Fig. 2). *S. marcescens was* isolated from patients who suffered sepsis belonging to the clonal groups A, C, and G, coinciding with those circulating in the NICU in that period. Figure 3 shows the temporary evolution of the clonal groups of *S. marcesc*ens. In general, there is no overlapping of the different clones at the time, except in March 2021, where four different clones coexist (Fig. 3).

### Follow-up and clinical impact

**Fig. 4:**
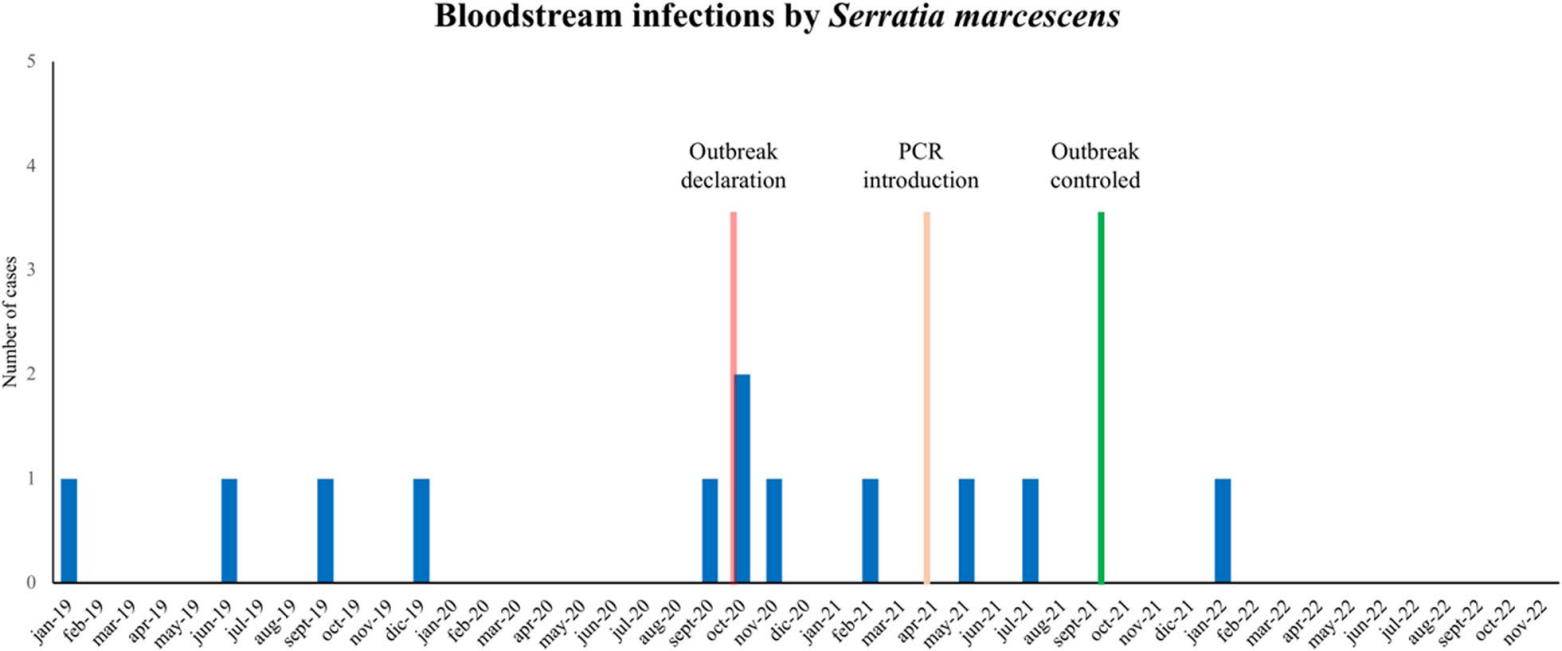
Cases of bloodstream infections caused by *S. marcescens* in the NICU in the pre-PCR and PCR periods

Eighteen months after the implementation of this new PCR, no more outbreaks by *S. marcescens* have been detected in the NICU of our hospital. Regarding the bloodstream infections by *S. marcescens*, taking a period of 33 months (from January 2019 until September 2021, when the outbreak was declared solved), a total of 11 cases of bloodstream infections by *S. marcescens* in neonates occurred. In contrast, only one case of bloodstream infection occurred from October 2021 until November 2022 (14 months) (Fig. 4).

## Discussion

In this study, we report how a molecular strategy based on PCR can be a powerful tool to improve the control of a nosocomial outbreak by *S. marcescens* in a NICU. The epidemiological analysis of the isolates revealed that 7 clonal groups of *S. marcescens* were circulating during the same outbreak (Fig. 3). This kind of behavior has been described previously in *S. marcescens* outbreaks [3, 6, 12, 13], where several clones are normally implied. Furthermore, the fact that patients were infected by the clonal group circulating at that moment, allows us to believe that clonal groups do not have a particular virulence.

Regarding the environmental study, in 6 out of the 15 samples where the DNA of *S. marcescens* was detected, the microorganisms could be isolated. In the other 9 samples, *S. marcescens* were not isolated, even after previous enrichment, suggesting the presence of DNA without viable microorganisms. Looking at those six *S. marcescens* isolates, 2 of them belong to the same clonal group (F), which was not detected infecting/colonizing any patients and the other 4 belong to 3 different clonal groups (A, B, and E) (Fig. 2). In view of these circumstances is very difficult to establish a common reservoir for this outbreak, suggesting a multicausal origin as reported previously [6, 14]. Regardless of the impossibility of clarifying the epidemiological origin of the outbreak, it seems that the implementation of this PCR-based strategy in the routine screening, along with the control measures adopted, has had a high clinical impact, decreasing considerably the bloodstream infections in the NICU. However, a prospective study over a longer period would be necessary to confirm whether this molecular strategy has clinical utility beyond outbreak control.

Focusing on the detection method, no standard screening strategy is recommended for detecting patients colonized by *S. marcescens* currently. Giles et al. [15] performed a study looking for the best screening method to detect *S. marcescens* during an outbreak in the NICU. They reported that a culture-based strategy implemented along with sample enrichment may increase profitability. However, these kinds of techniques are less sensitive than molecular methods. In this sense, another approximation based on Whole Genome Sequencing (WGS) of *S. marcescens* has been proposed by Muyldermans et al. [16]. Although this kind of strategy could be promising to map the spread of an outbreak with high power of resolution, the main problem is that prior to the sequencing, *S. marcescens* must be isolated in an agar plate, consequently not allowing rapid detection of the colonized patients. Furthermore, the use of WGS must always be supported by microbiological and epidemiological data in order to avoid errors [17]. To our knowledge, this is the first study reporting and evaluating the implementation of a PCR to detect patients colonized by *S. marcescens* in a NICU. Considering the results of our study, we think that adding a first step based on PCR detection of *S. marcescens* may accelerate the microbiological detection, reducing the time until the patient’s isolation and helping in the rapid outbreak control.

## Conclusions

In conclusion, the implementation of a PCR specific for *S. marcescens* detection has proved to be an excellent strategy to control the nosocomial spread of *S. marcescens* in the NICU. This kind of strategy, along with the rapid establishment of control measures, could help accelerate the resolution of nosocomial outbreaks and avoid severe infections by this microorganism and others.

### Supplementary Information

**Additional file 1: Figure S1.** Uncropped full-length agarose gels from PFGE. *Serratia marcescens* IDs are indicated above each lane. *Samonella braenderup* was used as standard. Empty lanes correspond to isolates excluded from this study.

## Data Availability

The datasets used and/or analysed during the current study are available from the corresponding author on reasonable request.

## Abbreviations

NICU: Neonatal Intensive Care Unit
BSI: Bloodstream infections
PFGE: Pulse field gel electrophoresis
WGS: Whole genome sequencing
