# Supplementary material for: Implementation of a PCR-based strategy to control an outbreak by Serratia marcescens in a Neonatal Intensive Care Unit
Source: Ann Clin Microbiol Antimicrob. 2023 Dec 11;22:108. doi: 10.1186/s12941-023-00657-0 (PMC10714559; doi:10.1186/s12941-023-00657-0)
Supplement: Supplementary file 1 — Additional file 1: Figure S1. Uncropped full-length agarose gels from PFGE. Serratia marcescens IDs are indicated above each lane. Samonella braenderup was used as standard. Empty lanes correspond to isolates excluded from this study. [file 12941_2023_657_MOESM1_ESM.pdf]

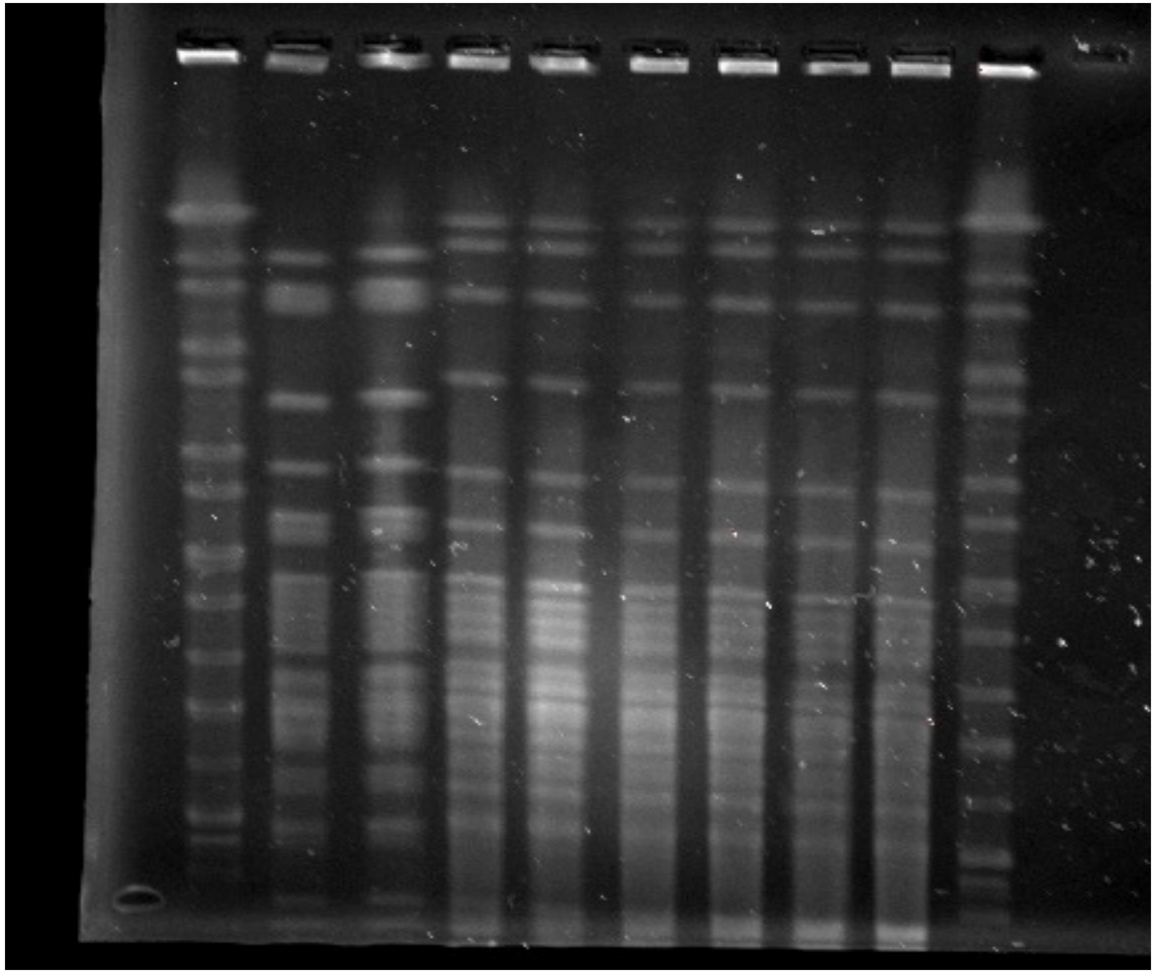
Salmonella braenderup
S3
S4
S5
S6
S7
S8
S9
S10
Salmonella braenderup

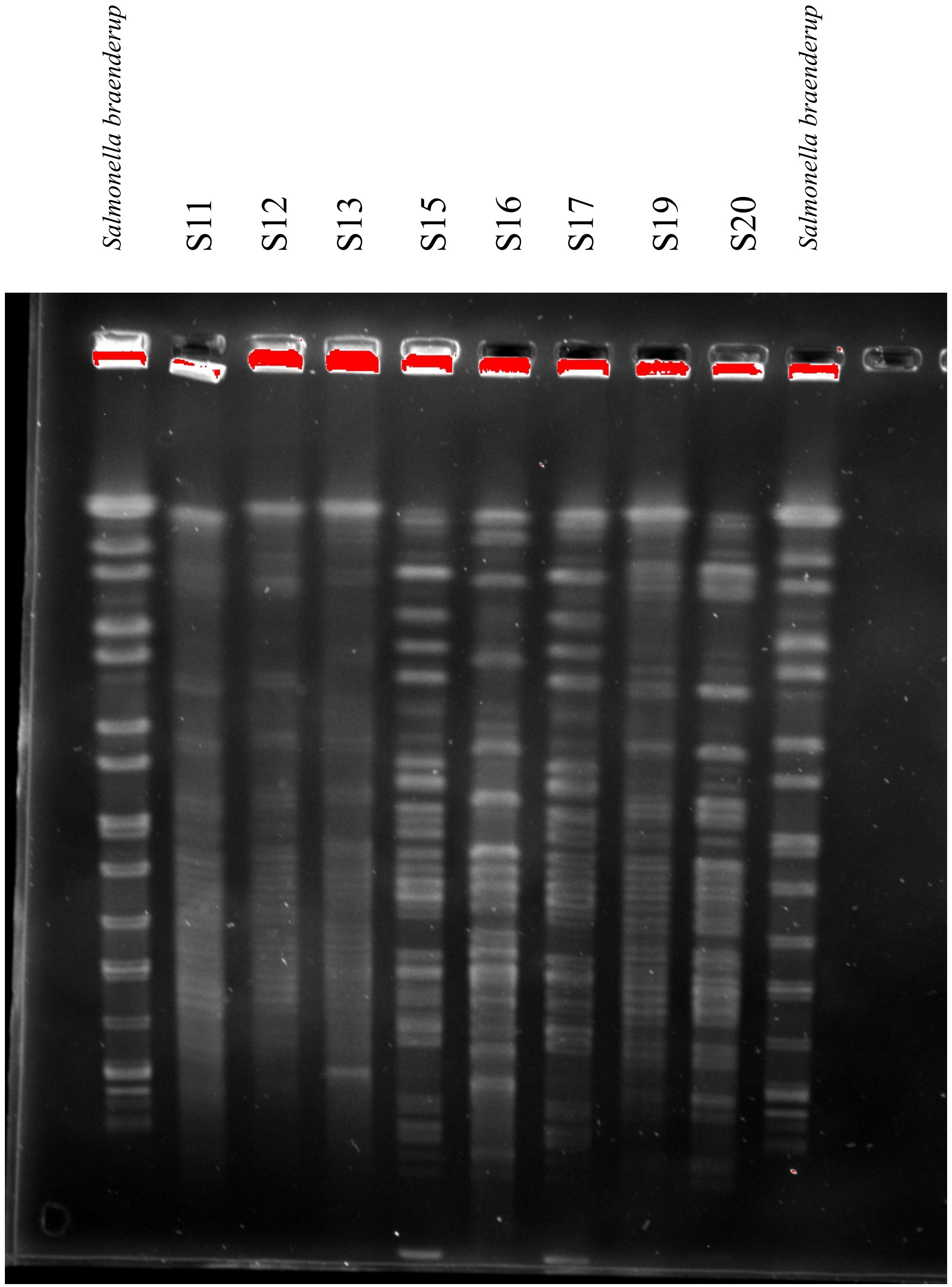

Salmonella braenderup
S11
S12
S13
S15
S16
S17
S19
S20
Salmonella braenderup

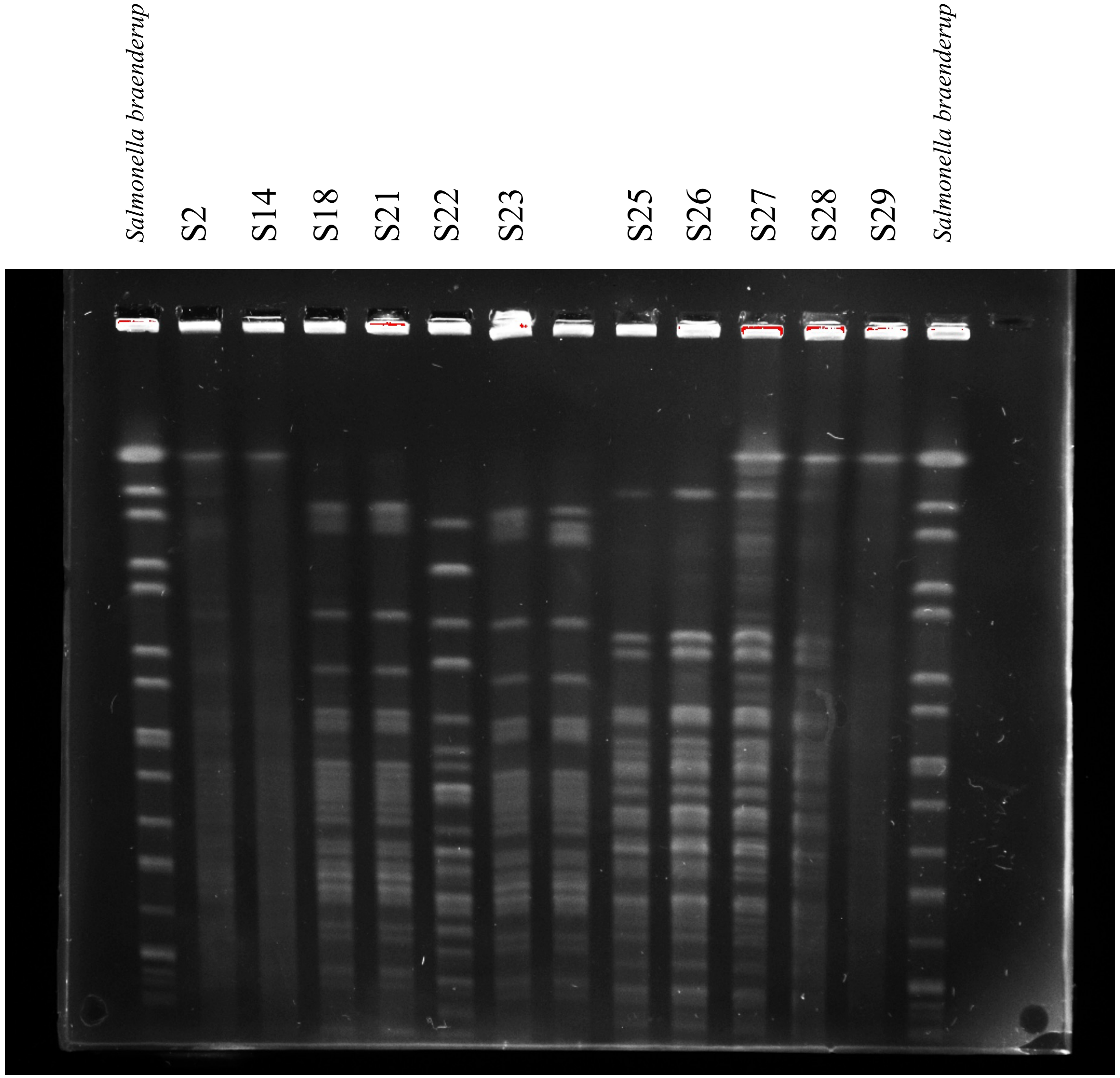
Salmonella braenderup
S2
S14
S18
S21
S22
S23
S25
S26
S27
S28
S29
Salmonella braenderup

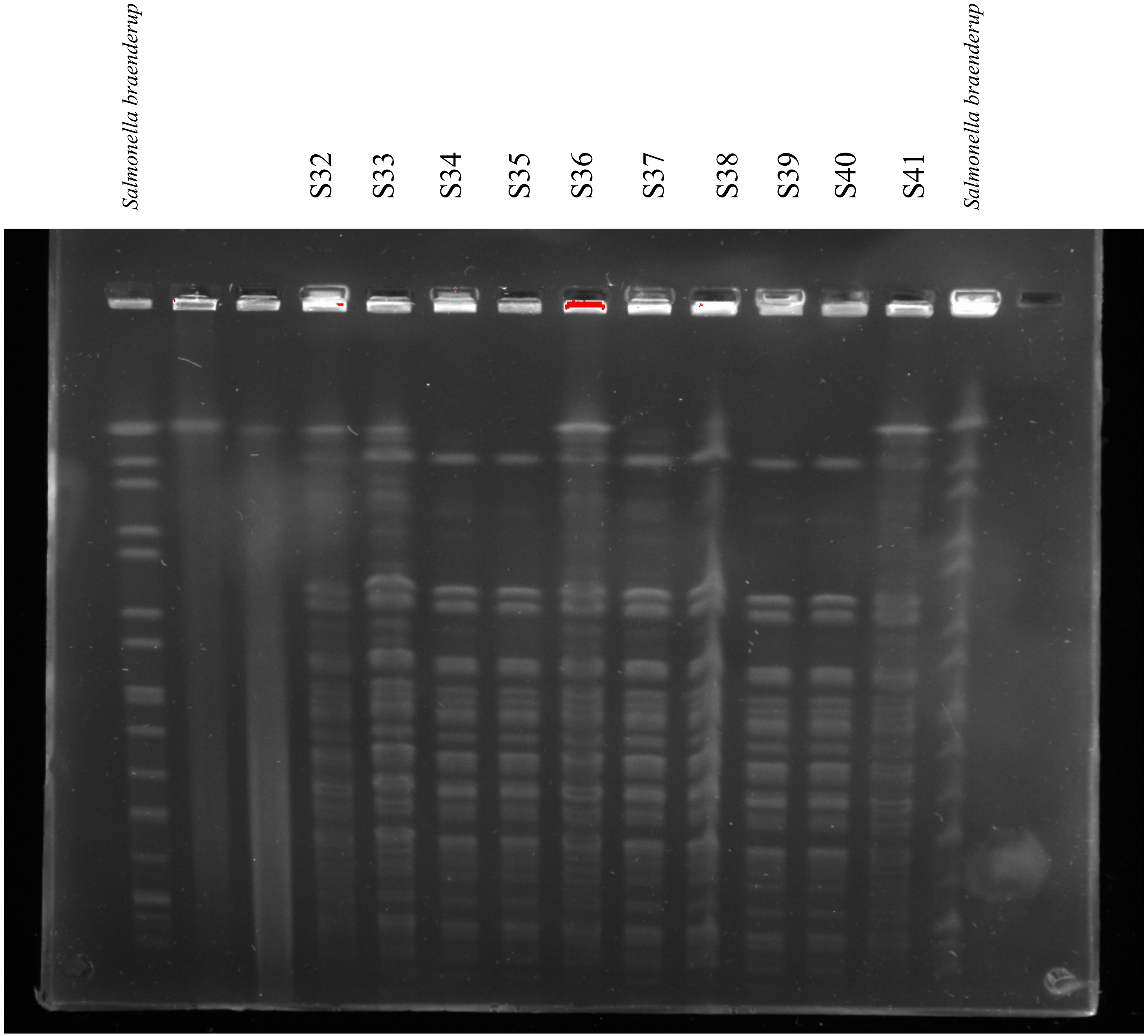

Figure S1. Uncropped full-length agarose gels from PFGE. *Serratia marcescens* IDs are indicated above each lane. *Samonella braenderup* was used as standard. Empty lanes correspond to isolates excluded from this study.
